# Characterizing the cytoprotective activity of Sarracenia purpurea L., a medicinal plant that inhibits glucotoxicity in PC12 cells

**DOI:** 10.1186/1472-6882-12-245

**Published:** 2012-12-05

**Authors:** Cory S Harris, Muhammad Asim, Ammar Saleem, Pierre S Haddad, John T Arnason, Steffany AL Bennett

**Affiliations:** 1 Neural Regeneration Laboratory and Ottawa Institute of Systems Biology, Department of Biochemistry, Microbiology, and Immunology, University of Ottawa, Ottawa, Canada; 2 Centre for Research in Biopharmaceuticals and Biotechnology, Department of Biology, University of Ottawa, Ottawa, ON, Canada; 3 Centre for Indigenous Peoples’ Nutrition and Environment School of Dietetics and Human Nutrition McGill University, Ste. Anne de Bellevue, Québec, Canada; 4 Natural Health Products and Metabolic Diseases Laboratory, Department of Pharmacology, Université de Montréal and Montreal Diabetes Research Center, Montréal, QC, Canada

**Keywords:** Diabetic neuropathy, Glucose toxicity, Traditional medicine, Quercetin-3-O-galactoside, Morroniside

## Abstract

**Background:**

The purple pitcher plant, *Sarracenia purpurea* L., is a widely distributed species in North America with a history of use as both a marketed pain therapy and a traditional medicine in many aboriginal communities. Among the Cree of Eeyou Istchee in northern Québec, the plant is employed to treat symptoms of diabetes and the leaf extract demonstrates multiple anti-diabetic activities including cytoprotection in an *in vitro* model of diabetic neuropathy. The current study aimed to further investigate this activity by identifying the plant parts and secondary metabolites that contribute to these cytoprotective effects.

**Methods:**

Ethanolic extracts of *S. purpurea* leaves and roots were separately administered to PC12 cells exposed to glucose toxicity with subsequent assessment by two cell viability assays. Assay-guided fractionation of the active extract and fractions was then conducted to identify active principles. Using high pressure liquid chromatography together with mass spectrometry, the presence of identified actives in both leaf and root extracts were determined.

**Results:**

The leaf extract, but not that of the root, prevented glucose-mediated cell loss in a concentration-dependent manner. Several fractions elicited protective effects, indicative of multiple active metabolites, and, following subfractionation of the polar fraction, hyperoside (quercetin-3-*O*-galactoside) and morroniside were isolated as active constituents. Phytochemical analysis confirmed the presence of hyperoside in the leaf but not root extract and, although morroniside was detected in both organs, its concentration was seven times higher in the leaf.

**Conclusion:**

Our results not only support further study into the therapeutic potential and safety of *S. purpurea* as an alternative and complementary treatment for diabetic complications associated with glucose toxicity but also identify active principles that can be used for purposes of standardization and quality control.

## Background

The purple pitcher plant, *Sarracenia purpurea* L. (Sarraceniaceae), is a perennial carnivorous plant widely distributed across northern North America. By consuming nitrogen from insects trapped within their pitchers (fused leaves), they adapt to nitrogen-poor environments such as bogs and peatlands. Due to this unusual natural history, *S. purpurea* has received considerable attention from an ecological perspective but, despite a long history of use as a traditional medicine across the continent, the therapeutic potential of the species remains largely uninvestigated. During the 19^th^ century, *S. purpurea* served as a treatment for small pox [1,2] and, more recently, as an injected pain reliever marketed as Sarapin®, an alkaline extract of the root that specifically blocks C-fibre excitation [3,4]. In Canada, the plant has long been recognized among aboriginal peoples as medicinal. The Cree of Eeyou Istchee (CEI) in Northern Québec refer to *S. purpurea* as “aygadash”, which translates to ‘frog’s socks’ in reference to the plant’s long slender pitchers and identify preparations from the leaves as beneficial in treating symptoms of diabetes, in particular slow healing infections [5].

The CEI, along with other Canadian First Nations communities, are recognized as some of the highest at-risk populations for T2DM in the world [6,7]. With an average age at diagnosis of just 41 years [8], diabetics also face a greater risk of developing diabetic complications [9,10]. As part of a collaborative research initiative evaluating traditional Cree medicines as culturally relevant treatment options for T2DM, our team tested a collection of plants, including *S. purpurea*, used by the CEI to treat symptoms of diabetes in a battery of anti-diabetic assays [11-14]. In addition to insulinotrophic effects in C2C12 muscle cells and 3T3-L1 adipocytes, the ethanolic extract of *S. purpurea* leaf material demonstrated cytoprotective activity in two models of diabetic neuropathy, PC12 cells exposed to glucose toxicity or glucose deprivation [11].

Currently, evidence supports the involvement of high, low, and fluctuating glucose concentrations in the pathophysiology of diabetic peripheral neuropathy [15-17]. Whereas hypoglycemia likely contributes to the development of diabetic neuropathy in Type 1 diabetics and T2DM patients on intense pharmacotherapy, hyperglycemia is likely the main contributor in populations such as the CEI where T2DM is far more prevalent and compliance to modern treatment regimens is generally low [9]. As such, culturally acceptable treatment options could benefit the control of glucotoxic neuropathic complications within the community. In this study, we sought to identify the plant organ sources and the active constituents contributing to the cytoprotective effects of *S. purpurea* under conditions of glucose toxicity. In comparing activities of root and leaf extracts, we demonstrate enhanced cytoprotective activity in the leaf extract as predicted by the traditional usage. Through subsequent bioassay-guided fractionation and phytochemical analyses, we identified and quantified marker compounds including biologically active metabolites contributing to cytoprotection.

## Methods

### Reagents

All cell culture reagents were obtained from Invitrogen (Burlington, ON Canada) and all chemicals were purchased through Sigma-Aldrich (St. Louis, USA) unless otherwise stated. Pure hyperoside (quercetin-3-*O*-galactoside), isoquercetin (quercetin-3-*O*-glucoside), and (-) epicatechin standards were purchased from Extrasynthèse (Lyon, France). Morroniside was isolated in-house to a purity of over 95% as determined by ultraviolet (UV) absorption, mass spectrometry (MS) and nuclear magnetic resonance (NMR) analyses.

### Plant materials and extracts

In August 2006, wild samples were collected near Mistissini, Quebec, Canada, as per the instructions of community elders and healers. The specimens were identified as *Sarracenia purpurea* L. by Dr. A. Cuerrier (Plant Biology Research Institute, Montréal Botanical Garden) and voucher specimens were deposited in the Marie-Victorin herbarium (MT) of the Montréal Botanical Garden. Upon collection, loose debris (such as peatmoss) was removed from the plants, which were subsequently partially dried by air (25°C) and transported to the University of Ottawa. Whole plants were separated into leaves (pitchers), roots, and flowers. Leaves were cut open and rinsed clean of insects and dirt. Leaf and root tissues were then dehydrated using an electric food dehydrator (Nesco/American Hervest WI, USA) set to 40°C and processed using a Wiley Mill (2 mm filter) prior to extraction with 80% ethanol as previously described [18]. Dried extracts were prepared for experimental use as stock solutions in dimethyl sulfoxide (DMSO), filtered through a 0.2 μm nylon membrane filter (Chromatographic Specialties Inc., Brockville, ON, Canada), and serially diluted as required on the day of use to ensure all cultures were exposed to a final concentration of 0.1% DMSO (vehicle).

### Cell culture and glucose toxicity assay

PC12-AC cells, a clonal derivative of the PC12 rat adrenal pheochromocytoma cell line (American Tissue Culture Collection) developed in our laboratory [19], were cultured in Roswell Park Memorial Institute medium (RPMI 1640) containing 11 mM glucose and supplemented with 10% horse serum and 5% newborn calf serum. Prior to experimental use, cells were seeded in 96-well plates at a density of 6.25x10^3^ cells/well and incubated overnight at 37°C in 5% CO_2_ to allow adherence. As described previously [18], extract toxicity (IC_50_) was determined by treating cultures for 96 h in serum-free RPMI 1640 containing 11 mM glucose, 0.025% bovine serum albumin (BSA) to facilitate intracellular passage of hydrophobic compounds, and either 0.1% DMSO (vehicle control) or increasing concentrations of plant extract (0-100 μg/mL). Similarly, for the glucose toxicity assay, cells were treated for 96 h in serum-free medium supplemented with glucose (to a final concentration of 150 mM), 0.025% BSA, and vehicle control or various concentrations of plant extract below its determined IC_50_ value.

### Cell viability assay

To assess viable cell number, the formazan dye WST (Roche Diagnostics, Laval, QC) was added to each well following 96 h of treatment in normal (11 mM) or high glucose (150 mM), as described by Harris et al. [18]. After a 60 min incubation with WST, spectrophotometric analysis at 420 nm (formazan) and at 620 nm (reference) was performed using a Tecan Spectra Shell platereader model A-5082 (Durham, NC) and WinSelect software (Tecan US, Inc.). Viable cell number per well was calculated relative to standard curves produced from wells containing known cell density present on each plate. All treatments were tested in multiple wells over two or three independent experiments (n = 8-15/condition). Data from vehicle-treated control cultures in normal and high glucose were included on all plates and combined across plates when applicable (n = 12-24). Percent viability was calculated as follows:

$$\%\text{viability}=\frac{{\text{cell number}}_{\left(\text{treatment well}\right)}}{{\text{mean cell number}}_{\left(\text{normoglucose control}\right)}}\times 100$$

### Cell survival assay

Cell survival was directly assessed by Live/Dead viability/cytotoxicity assay (Invitrogen) as described previously [20-22]. Viable cells, identified by the conversion of non-fluorescent calcein-AM to fluorescent calcein by intracellular esterases, and dead cells, identified by nuclear incorporation of cell-impermeable ethidium homodimer (ET), were imaged using a DMIR epifluorescent inverted microscope (Leica, Richmond Hill, Canada) coupled with a QICAM digital camera (Quorum Technologies, Guelph, Canada). Images were captured and analysed using OpenLab software v5.05 (Improvision, Lexington, USA). Percent survival was calculated as:

$$\begin{array}{l} \%\;\text{survival}\\ \;=\frac{{\text{Viable cell number per well}}_{\left(\text{calcein-positive and ET-negative}\right)}}{{\text{Mean viable cells in vehicle control}}_{\left(\text{calcein-positve and ET-negative}\right)}}\\ \;\times \;100 \end{array}$$

### Phytochemical characterization

The total phenolic content of root and leaf extracts was determined using the Folin-Ciocalteau method described previously [23,24]. Total phenolic content, calculated relative to serially diluted quercetin standard analyzed concurrently with extracts, were expressed as quercetin equivalents. Chromatographic analyses of root and leaf extracts were performed on an Agilent 1100 high pressure liquid chromatography (HPLC) system (Palo Alto, CA, USA) comprised of an autosampler equipped with a 100 μL loop, a quaternary pump with maximum pressure of 400 bars, a column thermostat, a photodiode array detector (DAD), and an online atmospheric pressure chemical ionization mass selective detector (APCI/MSD VL 1946C). Separations were executed using a validated method on a YMC ODS-AM column (100 × 2 mm I.D.; 3 μm particle size) (Distributed by Waters Inc., Mississauga, Canada) as previously described [25] with minor modifications. Using aqueous trifluoroacetic acid (TFA, 0.05%), pH 3.4 (solvent A) and methanol (solvent B) as mobile phase, initial conditions 92%:8% (A:B) were held for 2 min followed by four linear gradients of 8 – 13% B in 3 min, 13 – 30% B in 15 min, 30 – 60% B in 5 min, and 60 – 100% B in 4 min. Solvent composition was then returned to initial conditions, which were maintained for 7 min to allow re-equilibration. Solubilized samples were filtered through 0.2 μm PTFE membrane filter (Chromatographic Specialities Inc., Brockville, ON) prior to analysis. Chromatographic separations were monitored at 325 nm, 230 nm and at 520 nm (band width 4, reference off) by DAD and MSD detection was performed in positive ionization scan mode as optimized previously [25].

Initial compound identifications were performed by matching the UV spectra of eluted peaks with those of standards registered in an on-line Chemstation library. Confirmation of identity was achieved through comparison of fragmentation patterns and relative retention times with those of reference standards and/or isolated compounds identified by NMR. Identified metabolites were quantified on the basis of area under the peak of DAD signals (at 230 nm for morroniside and 280 nm for epicatechin and hyperoside) using calibration curves produced with pure compounds analyzed on the same day (n=3).

### Isolation of active compounds

Prior to fractionation by low-pressure column chromatography, the crude leaf extract was washed with hexane to remove the lipophilic fraction then dried and solubilized in methanol. To separate the more polar compounds, distilled water was added to the methanolic solution and the resulting precipitate was collected by centrifugation (the methanol fraction). The remaining soluble fraction (water-methanol fraction) was dried by lyophilization. The methanol-water fraction was loaded onto a 100 × 5 cm Sephadex LH-20-packed glass column and separated using a step-wise gradient from 100% water to 100% methanol in 10% increments every 100 min. Collected samples were pooled into 10 fractions based on HPLC profiles, each of which was subsequently tested for cytoprotective activity. Subfractionation of active fractions was conducted using an Agilent 1200 Series semi-preparative HPLC with online DAD and automated time-based fraction collection. Following the subfractionation, additional purification of major peaks was achieved by re-eluting the collected subfractions using peak-based fraction collection. The identity of marker compounds was confirmed by ^1^H-NMR and ^13^C-NMR then, in the case of hyperoside, morroniside, and epicatechin, confirmed by comparing retention time, UV and MS data to those of purified standards.

### Statistical analyses

Data were analyzed using one-way ANOVA tests followed by *post hoc* Dunnett’s *t*-tests for each extract, fraction, or pure compound relative to normal and high glucose controls, respectively. Differences with *p* values less than 0.05 were considered statistically significant and were represented as * (relative to normal glucose control) or # (relative to high glucose control). *P* values under 0.01 were considered highly significant (shown as ** or ##).

## Results and discussion

Prior to evaluating protective effects, the toxicities of *S. purpurea* leaf and root extracts were established following 96 h exposure to cells in serum-free, normal glucose (11 mM) conditions. IC_50_ concentrations were defined as the extract concentration eliciting a 50% loss of viable cells relative to control cultures. Cell viability was determined by mitochondrial dehydrogenase cleavage of the formazan dye WST relative to vehicle-treated (0.1% DMSO) control cells. Both extracts were well-tolerated by cells but, with IC_50_ concentrations of 129 μg/mL and 56 μg/mL, respectively, the leaf extract was less toxic than that of the root.

**Figure 1:**
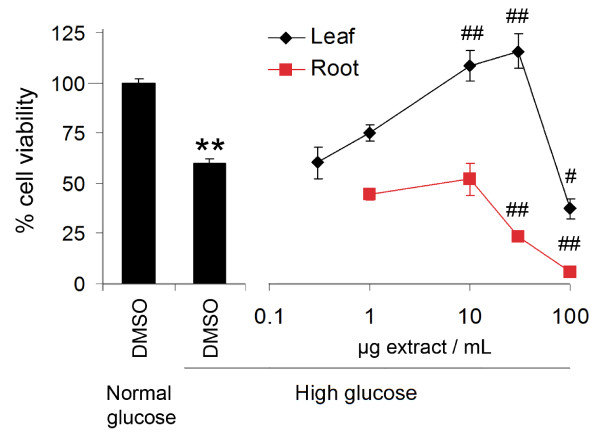
**Leaf but not root extract of *Sarracenia purpurea *protects PC12 cells from high glucose-mediated death.** Exposure to glucose toxicity elicited a significant loss in cell viability compared to normal glucose conditions as assessed by mitochondrial dehydrogenase activity measured by cleavage of the formazan dye WST [left panel, ** denotes a significant difference (*p* < 0.01) relative to normal glucose control, Student’s *t*-test, n = 24]. Multiple concentrations of leaf or root extract were administered to high glucose-treated cells to assess cytoprotective activity [right panel, # and ## denote significant differences (*p* < 0.05 and ## *p* < 0.01, respectively) relative to high glucose control, ANOVA, post-hoc Dunnett’s *t*-test, n = 8-12]. Data are reported as the mean ± SEM. Extracts were tested at concentrations below their IC_50_ concentrations established under normal glucose conditions.

PC12-AC cells can be differentiated to a peripheral catecholaminergic neuron phenotype by the combination of serum deprivation and treatment with nerve growth factor. As such, these cells have commonly been used to model neuronal stress and serve as an accessible model of diabetic peripheral neuropathy repeatedly used by us and others [11,26-28]. Consistent with previous reports using the current protocol [29], the viability of vehicle-treated PC12-AC cells exposed to elevated glucose concentrations (150 mM) for 96 h was reduced by 40-50% relative to vehicle-treated cells under normal glucose conditions (Figure 1, left panel). This cell loss is glucose-specific and not due to osmotic stress as substitution of d-glucose for l-glucose abolishes toxicity [18,30]. Other studies have demonstrated a similar loss (30%) of PC12 cells exposed to 75 mM high glucose media once differentiated to a neuronal phenotype [28]. To ascertain whether the protective activity of *S. purpurea* in the glucotoxicity model [11] is organ-specific, root and leaf extracts were evaluated at various concentrations below their respective IC_50_ concentrations (0-30 μg/mL for root and 0-100 μg/mL for leaf). The leaf extract reduced glucose-induced cell loss in a concentration-dependent manner up to 30 μg/mL but failed to provide protection at 100 μg/mL. Conversely, the root had no appreciable effect on glucose toxicity at low concentrations but significantly exacerbated cell loss when concentrations approached the IC_50_ value (Figure 1, right panel).

**Figure 2:**
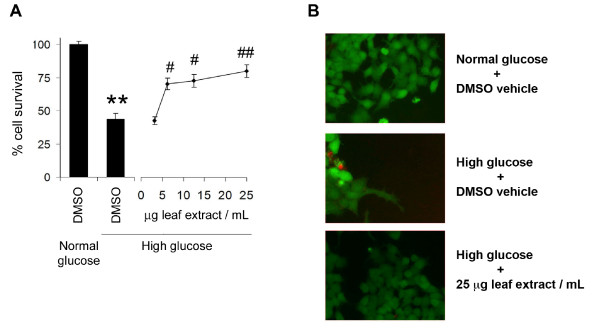
**Leaf extract of *Sarracenia purpurea *prevents high glucose-mediated cytotoxicity.** (**A**) Exposure to glucose toxicity elicited a significant loss in cell survival compared to normal glucose conditions as assessed by Live/Dead assay [left panel, ** denotes a significant difference (*p* < 0.01) relative to normal glucose control, Student’s *t*-test, n = 8]. Multiple concentrations of leaf extract were administered to high glucose-treated cells to confirm cytoprotection [right panel, # and ## denote significant differences (*p* < 0.05 and ## *p* < 0.01, respectively) relative to high glucose control, ANOVA, post-hoc Dunnett’s *t*-test, n = 8]. (**B**) Treatment with 25 μg/mL of leaf extract reversed high glucose-induced loss of PC12 cells, which were identified by intracellular cleavage of calcein-AM to its fluorescent product (green cells) without loss of membrane integrity (red cells). Data are reported as the mean ± SEM.

In order to confirm the cytoprotection offered by *S. purpurea* leaf extracts, a more direct measure of cell survival was employed since mitochondrial dehydrogenase activity can be either elevated in dying cells or reduced in metabolically compromised but not terminally damaged cells thereby confounding readouts of WST absorbance [31]. For this reason, we refer to cell number determined using the WST assay as viable cell number compared to other measures of cell survival. Following the same treatment protocol, serum-deprived cultures were treated with 0.1% DMSO (control) or increasing concentrations of leaf extract. Cell survival was subsequently quantified by Live/Dead assay after 96 h. This technique allows for direct assessment of viable and dead (or dying) cells in culture as well as cells that have detached over the course of treatment; viable adherent cells were identified by cleavage of calcein AM to its fluorogenic product by intracellular esterases and dead or dying cells were identified by uptake of the membrane-impermeant ethidium bromide homodimer. Data are expressed relative to surviving cell number in vehicle-treated cultures in normoglucose to account for cell loss over treatment. Upon exposure to high glucose, cell survival in vehicle-treated cultures was compromised by more than 50% (Figure 2A,B). Cell loss was significantly inhibited by the presence of leaf extract with concentration-dependent protection (Figure 2A,B). As observed by WST determination, exposure to leaf extract (0-25 μg/mL) did not affect the survival of cells under serum-free normal glucose conditions (data not shown).

**Figure 3:**
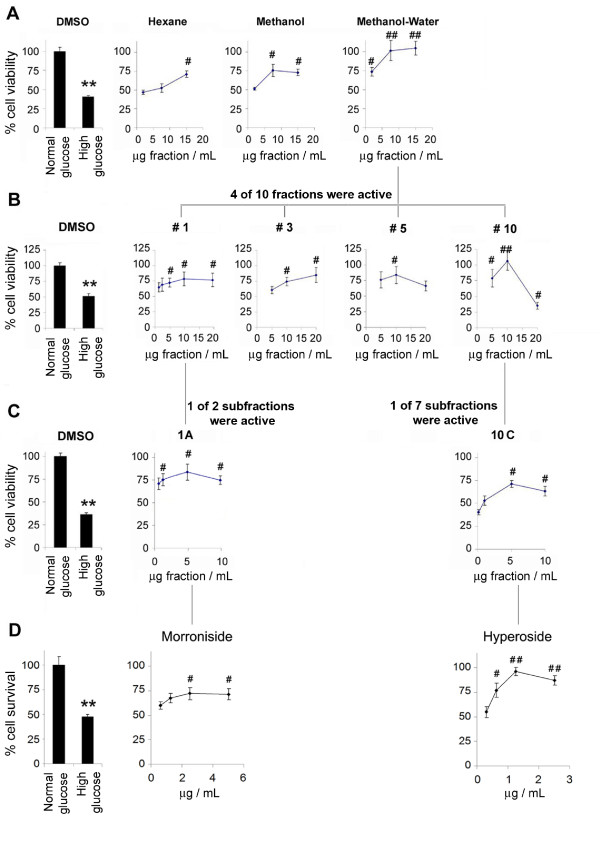
**Activity-guided fractionation of *Sarracenia purpurea *leaf extract.** In the first phase the crude leaf extract was fractionated based on solubility in hexane, methanol and water. (**A**) Each of the solvent fractions was evaluated in the glucose toxicity cell viability assay using WST and the methanol-water fraction was selected for further fractionation by Sephadex LH-20 column chromatography. (**B**) Each of the subsequent fractions was similarly evaluated with four of ten demonstrating significant cytoprotection. Fractions #1 and #10 were selected for further fractionation by semi-preparative HPLC. (**C**) Upon evaluation of resulting subfractions using the WST assay, only subfractions #1A and #10C, significantly reduced cell loss. (**D**) The concentration dependent protective activities of pure morroniside (>95%), the predominant metabolite in fraction #1, and hyperoside (>95%), the predominant metabolite in subfraction #10C, were confirmed using the Live/Dead cell survival assay. In left panels, ** denotes a significant difference (*p* < 0.01) relative to normal glucose control (Student’s *t*-test, n = 8-12). In right panels, # and ## denote significant differences (*p* < 0.05 and ## *p* < 0.01, respectively) relative to corresponding high glucose control (ANOVA, post-hoc Dunnett’s *t*-test, n = 8-12.

The 80% ethanol extraction of dry leaf material produced a yield of 24.4%. As an initial fractionation step, lipophilic substances were separated into the ‘hexane’ fraction, which represented 6.5% of the crude extract by weight. The resulting defatted fraction was difficult to solubilize at high concentration (>30 mg/mL) in methanol and precipitated significantly with the introduction of water. The concentrated methanolic extract was therefore mixed with an equal part of cold water that, after centrifugation, was separated in two fractions, the precipitate (methanol fraction) consisting of over 30% by weight of the crude extract and the supernatant (methanol-water fraction). The dried hexane, methanol and methanol-water fractions were each dissolved in DMSO and assessed for protective effects in high glucose treated cultures using the WST viability assay (Figure 3A). Remarkably, all three extracts significantly improved cell viability in high glucose media in a concentration-dependent manner (3-15 μg/mL) (Figure 3A).

Because the methanol-water fraction was the most active, represented the largest fraction of the crude extract, and is likely most similar in chemical content to traditional Cree preparations, this fraction was selected for further fractionation by Sephadex LH20 column chromatography. When tested in the high glucose paradigm, 4 of the 10 subfractions significantly improved cell viability (Figure 3B, results from inactive fractions not shown). Of these, fractions 3 and 5 were of low yield whereas fractions 1 and 10 were the two largest by weight (29% and 9.0% of the crude extract, respectively).

Fraction 1 (10% MeOH) contained a large amount of saccharides with a single major peak detected by HPLC-DAD/APCI-MS. Once separated by preparative HPLC using peak-based fraction collection, this peak was identified as morroniside, an iridoid glycoside previously reported in the genus [32], by ^1^H and ^13^C NMR. Eliciting a similar response as the original fraction (#1), morroniside was subsequently purified and confirmed as the active component by WST (cell viability, Figure 3C) and Live/Dead (cell survival, Figure 3D) assays. Because the HPLC trace of fraction #10 (100% MeOH) was fairly complex, a third round of fractionation was performed by semi-preparative HPLC with automated fraction collection. Using time-based peak collection, seven subfractions (10A-G) were collected and prepared for administration to PC12-AC cells. As presented in Figure 3C, only subfraction 10C significantly reduced glucose-induced cell death.

HPLC-DAD/APCI-MS analysis of subfraction 10-C revealed a single peak accounting for > 90% of the total area under the chromatograph with UV and MS data consistent with a quercetin monoglycoside. Using pure quercetin standards as references, in-house library matching and ^1^H NMR, the unknown peak was identified as hyperoside (quercetin-3-*O*-galactoside). Apart from hyperoside, other quercetin glycosides are also known to occur in the species [33]. To verify that this compound was indeed responsible for the cytoprotective effects elicited by the subfraction, a commercially purchased hyperoside standard was tested in the PC12 model, significantly improving survival as determined by Live/Dead assay (Figure 3D).

**Figure 4:**
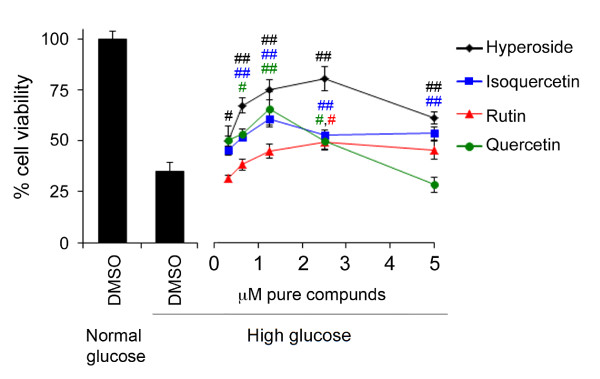
**Quercetin and its glycosides protect PC12 cells from high glucose-mediated death.** Exposure to glucose toxicity elicited a significant loss in cell viability compared to normal glucose conditions as assessed by mitochondrial dehydrogenase activity measured by cleavage of the formazan dye WST [left panel, ** denotes a significant difference (*p* < 0.01) relative to normal glucose control, Student’s *t*-test, n = 16]. Multiple concentrations of quercetin aglycone, hyperoside, isoquercetin (quercetin-3-O-glucoside), and rutin (quercetin-3-O-rutinoside), were administered to high glucose-treated cells to assess cytoprotective activity [right panel, coloured # and ## denote significant differences (*p* < 0.05 and ## *p* < 0.01, respectively) between high glucose control and similarly coloured samples, ANOVA, post-hoc Dunnett’s *t*-test, n = 8-10]. Data are reported as the mean ± SEM.

In previous studies using this model, plant extracts containing quercetin derivatives have yielded varying results. Whereas we reported that quercetin glycosides may contribute to organ-specific cytoprotective effects of *Vaccinium angustifolium* and *Picea glauca*[18,29], quercetin-containing extracts of *Vaccinium vitis-idaea* and *Kalmia angustifolia* were ineffective [12]. To address this inconsistency, different quercetin glycosides were assessed individually. Though quercetin and each of its glycosides showed similar responses with increasing activity to an approximate concentration of 5 μM before protection waned, the 3-*O*-galactoside (hyperoside) and 3-*O*-rutinoside were most and least effective, respectively (Figure 4). Within a given extract, both the specific moieties as well as the relative and absolute quantities of quercetin derivatives are thus likely to impact overall activity. It is important to note that, consistent with these results in undifferentiated PC12-AC cells, recent studies have also demonstrated protective activity of quercetin following challenge of PC12 cells differentiated to a neuronal phenotype with 75 mM high glucose [28].

**Figure 5:**
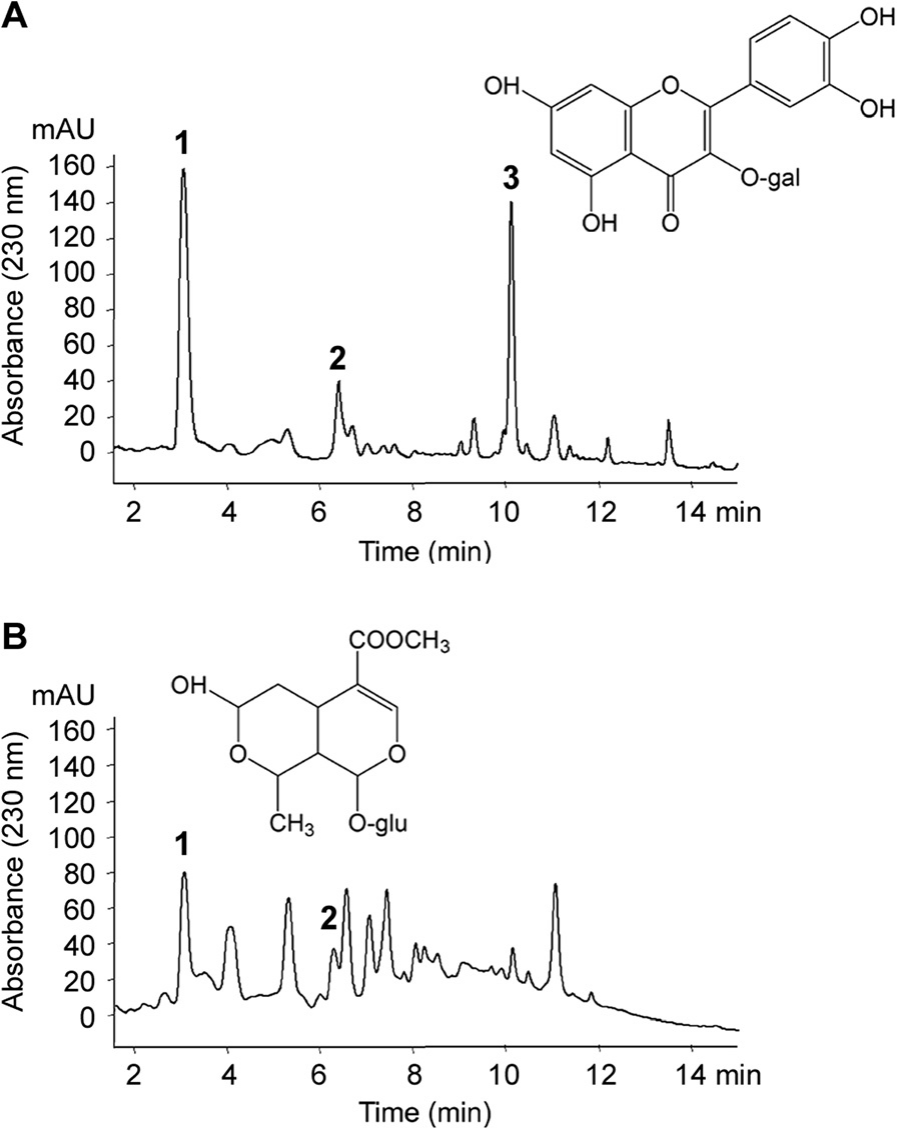
**HPLC chromatograms of *Sarracenia purpurea *leaf and root extracts.** Chromatograms of ethanolic leaf (**A**) and root (**B**) extracts with diode array detection at 230 nm wavelength. Labelled peaks represent the marker compounds morroniside (1), epicatechin (2), and hyperoside (3). The structures of the identified active metabolites (1**,** 3) are shown with quantitative results reported in Table 1 .

We next sought to characterize the crude leaf and root extracts to 1) determine whether the identified actives were present in sufficient concentrations to exert their effects, and 2) provide qualitative and quantitative data for quality control purposes. HPLC-DAD/APCI-MSD analysis revealed distinguishing differences between leaf and root extracts, most notably the absence of the large hyperoside peak with the retention time of 10 min in the root chromatograph (Figure 5). The concentration of hyperoside in the leaf extract, determined relative to a pure standard, was 62.5 μg/mL (Table 1). Three compounds, goodyeroside, epicatechin, and morroniside, were identified in both extracts and serve as species markers. Whereas epicatechin and morroniside were identified by UV and MS spectra and quantified relative to a pure standard, goodyeroside was identified by MS as well as ^1^H and ^13^C NMR spectra but was not quantified.

**Table 1:** **Quantitation of marker compounds in leaf and root extracts of *Sarracenia purpurea *by HPLC-DAD**

**Yield**^**a**^	**Leaf**		**Root**	
	**24.4%**		**14.6%**	
	**mg / g extract**	**mg / g dry plant tissue**	**mg / g extract**	**mg / g dry plant tissue**
1 – Morroniside	145.3 ± 1.1	35.5 ± 0.4	21.3 ± 0.6	3.1 ± 0.1
2 – Epicatechin	35.2 ± 1.8	8.6 ± 0.4	48.1 ± 1.7	7.0 ± 0.2
3 – Hyperoside	62.5 ± 1.0	15.3 ± 0.3	n/d	n/d
Goodyeroside	✓		✓	
Total phenolic content ^b^	74.5 ± 6.1	18.2 ± 1.5	146.1 ± 6.2	21.3 ± 0.9

Results are expressed as the mean of three replicates ± the standard error of the mean.

n/d = not detected.

^a^ Yield was calculated as (mass recovered extract / mass dry material) × 100%.

^b^ Expressed as mg quercetin equivalents / g extract as determined using with the Folin Ciocalteau method.

✓ Presence detected but not quantified.

At their detected concentrations within the leaf extract, both morroniside and hyperoside significantly reduced PC12-AC cell loss but neither was as active as the crude extract. Moreover, assay-guided fractionation revealed significant activity in multiple leaf fractions suggesting additive or synergistic effects that require further investigation to resolve. Recently, morroniside was isolated from Shan Zhu Yu (*Cornus officinalis* SIEB. et ZUCC.), a Traditional Chinese Medicine used for kidney problems [34]. In models of nephropathy and neurodegeneration, morroniside demonstrates strong anti-oxidant, reno- and neuroprotective effects [34-36] that likely contribute to Shan Zuh Yu’s beneficial properties.

With the previously known bioactivities ascribed to quercetin and its glycosides, the isolation of hyperoside as one of the active metabolites responsible for *S. purpurea*’s cytoprotective effects is not surprising. Our data are consistent with previous findings indicating that quercetin derivatives display anti-diabetic and neuroprotective activities relevant to the treatment or prevention of diabetic neuropathy [28]. Three of the major factors contributing to microvascular complications of diabetes, such as neuropathy, include oxidative stress, the formation of advanced glycation endproducts (AGEs), and increased flux through the polyol pathway [37,38]. As established anti-oxidants that inhibit both AGE formation [39] and aldose reductase activity [40], quercetin derivatives potentially act through a number of mechanisms. Although this study was not conducted in primary neurons, we have previously confirmed the neuroprotective activity of plant compounds identified through preliminary testing in PC12 cells [22] and recent studies have validated this finding in neuronally differentiated PC12 cultures [28]. Moreover, since quercetin and quercetin glycosides prevent neuronal death in several *in vitro* and *in vivo* models of neurodegeneration [41,42], their activity in the current model is promising.

The observed effects of the leaf extract are, however, of increased interest considering that Sarapin®, a root extract of *S. purpurea*, is used to relieve pain. Though clinical evidence supporting Sarapin® is incomplete and the putative active constituents remain unidentified, the preparation has been used for a variety of pain-related ailments [3,4]. With further study, *S. purpurea* products could potentially provide both symptomatic relief and slowed progression of diabetic neuropathy through the preparation of two separate medicines, a leaf tincture and an alkaline root extract.

## Conclusions

Our study provides compelling evidence that traditional preparations of *S. purpurea*, such as those used by CEI healers, are based on tangible pharmacological agents and actions *in vitro*. While restricted to cell-based assays, this provides further experimental support for the traditional use of the plant in CEI communities and has identified active metabolites – hyperoside and morroniside – that will guide future investigations into the extract’s mechanism(s) of action focusing on potential synergies as well as additional species markers valuable for quality assurance and standardization purposes.

## Abbreviations

AGE: Advanced glycation endproduct; APCI: Atmospheric pressure chemical ionization; BSA: Bovine serum albumin; DAD: Diode array detector; DMSO: Dimethylsulfoxide; DPPH: Diphenylpicrylhydrazyl; HPLC: High pressure liquid chromatography; IC50: Inhibitory concentration 50, concentration at which treatment elicits death of 50% of cells; MS: Mass spectrometer; MSD: Mass selective detector; m/z: Mass over charge ratio; NMR: Nuclear magnetic resonance; PTFE: Polytetrafluoroethylene; RPMI: Roswell Park Memorial Institute medium; SEM: Standard error of the mean; T2DM: Type 2 diabetes mellitus.

## Competing interests

The authors have no competing interests.

## Authors’ contributions

CSH conducted the cytoprotection assays, processed plant materials, carried out fractionations, performed phytochemical and statistical analyses, and drafted the manuscript. MA conducted NMR experiments and deciphered molecular structures. AS developed the methods of phytochemical analyses and assisted with fractionation. PSH, the project leader, conducts complementary research on *S. purpurea* and contributed meaningfully to the manuscript. JTA and SALB participated in designing the analytical and cell-based components of the study, respectively, and assisted with data analysis, interpretation, and wrote the manuscript with CSH. All authors read and approved the final manuscript.

## Pre-publication history

The pre-publication history for this paper can be accessed here:

http://www.biomedcentral.com/1472-6882/12/245/prepub
